# Common Non-Rheumatic Medical Conditions Mimicking Fibromyalgia: A Simple Framework for Differential Diagnosis

**DOI:** 10.3390/diagnostics14161758

**Published:** 2024-08-13

**Authors:** Andrea D’Amuri, Salvatore Greco, Mauro Pagani, Barbara Presciuttini, Jacopo Ciaffi, Francesco Ursini

**Affiliations:** 1 General Medicine Unit, Medical Department, ASST Mantova, Ospedale Carlo Poma, Str. Lago Paiolo 10, 46100 Mantova, Italy; andrea.damuri@asst-mantova.it (A.D.); mauro.pagani@asst-mantova.it (M.P.); barbara.presciuttini@asst-mantova.it (B.P.); 2 Internal Medicine Unit, Medical Department, Ospedale del Delta, Via Valle Oppio 2, Lagosanto, 44023 Ferrara, Italy; salvatore.greco@unife.it; 3 Medicine & Rheumatology Unit, IRCCS Istituto Ortopedico Rizzoli, 40136 Bologna, Italy; francesco.ursini2@unibo.it; 4 Department of Biomedical and Neuromotor Sciences, Alma Mater Studiorum University of Bologna, 40136 Bologna, Italy

**Keywords:** fibromyalgia, diagnosis, vitamin deficiency, sleep apnea, thyroid diseases, infections, cancer, drug-induced

## Abstract

Fibromyalgia (FM) is a chronic non-inflammatory disorder mainly characterized by widespread musculoskeletal pain, fatigue, sleep disturbances, and a constellation of other symptoms. For this reason, delineating a clear distinction between pure FM and FM-like picture attributable to other common diseases can be extremely challenging. Physicians must identify the most significant confounders in individual patients and implement an appropriate diagnostic workflow, carefully choosing a minimal (but sufficient) set of tests to be used for identifying the most plausible diseases in the specific case. This article discusses prevalent non-rheumatological conditions commonly observed in the general population that can manifest with clinical features similar to primary FM. Given their frequent inclusion in the differential diagnosis of FM patients, the focus will be on elucidating the distinctive clinical characteristics of each condition. Additionally, the most cost-effective and efficient diagnostic methodologies for accurately discerning these conditions will be examined.

## 1. Introduction

Fibromyalgia (FM) is a chronic non-inflammatory disorder mainly characterized by widespread musculoskeletal pain, usually described as a deep ache in muscles, throbbing, intense, and persistent, with generalized burning and tingling [1]. In most patients, the clinical picture extends well beyond pain and is characterized by a constellation of other symptoms such as fatigue, sleep disturbances, cognitive dysfunction, or depressed mood [1]. FM incidence is 7–9 times higher in females [1] with prevalence estimates ranging between 1% and 8% overall and a relevant economic strain on healthcare systems [2].

Despite the substantial social burden imposed by FM, the process of diagnosing it and implementing an effective treatment regimen remains challenging. This complexity arises from various factors, including the heterogeneity and lack of specificity of many of the symptoms, overlapping clinical presentations with other medical conditions, and the absence of specific biomarkers or diagnostic tests.

Although the 2016 revision of the FM diagnostic criteria [3] clearly emphasizes that *a diagnosis of fibromyalgia is valid irrespective of other diagnoses*, thereby categorizing FM as a “positive diagnosis”, it also acknowledges that *a diagnosis of fibromyalgia does not exclude the presence of other clinically important illnesses*. Consequently, the clinical pathway culminating in a diagnosis of FM typically implies anyway the active search for other potential diseases underlying the patient’s complex polysymptomatology, making FM a diagnosis of exclusion in real-world practice.

**Figure 1:**
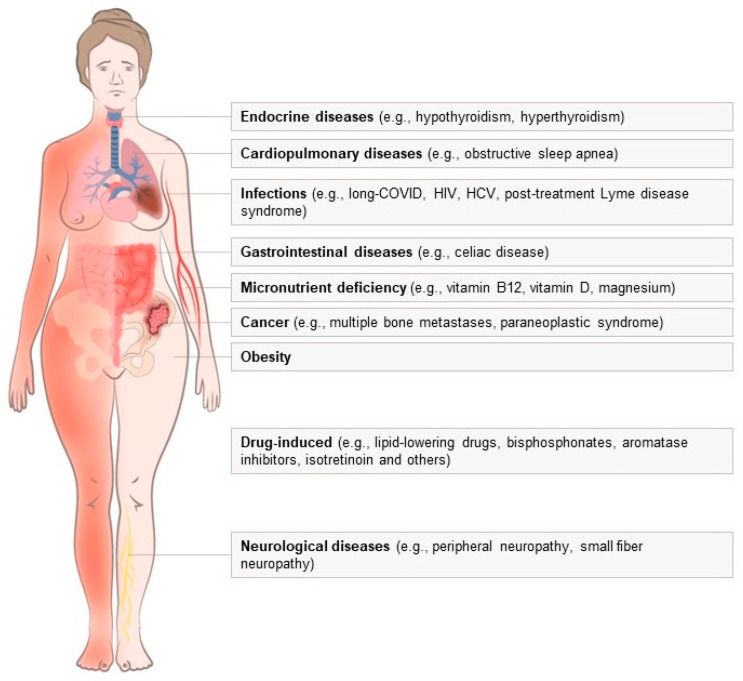
Schematic representation of the differential diagnoses of fibromyalgia.

Anyway, given the extensive array of differential diagnoses, delineating a clear distinction between pure FM and FM-like picture attributable to other diseases (Figure 1) can be extremely challenging. Therefore, physicians must identify the most significant confounders and implement an appropriate diagnostic workflow, carefully choosing a minimal (but sufficient) set of tests to be used for identifying the most plausible diseases in the specific case.

On this basis, the objective of this article is to delineate the spectrum of differential diagnoses that clinicians might erroneously attribute to FM.

In particular, the focus of the article will be elucidating the distinctive clinical characteristics and the diagnostic pathway of non-rheumatological conditions commonly observed in the general population, which can manifest with clinical features similar, at least in part, to that of primary FM. The decision to exclusively address non-rheumatologic differential diagnoses stems from the necessity to streamline the FM diagnostic process. Inflammatory rheumatic diseases, being typically identified and managed by rheumatologists through “robust” diagnostic tools (e.g., serum biomarkers, imaging), will be deliberately omitted from our discussion. By concentrating solely on non-rheumatologic differentials (Table 1), our objective is to provide clinicians with a more pragmatic resource for recognizing alternative conditions commonly encountered in patients with FM-like presentation in order to build a simple basic framework for differential diagnosis in primary care or orthopedics settings.

## 2. Micronutrient Deficiency

### 2.1. Vitamin B12 Deficiency

Vitamin B12, also called cobalamin, is a fundamental co-factor for nucleic acid and myelin synthesis [4]. In nature, vitamin B12 is synthesized by microorganisms, and humans obtain it solely through the consumption of animal-derived foods such as meat, eggs, or dairy products [4]. Cobalamin undergoes a complex absorption process, which involves the acidic environment of the stomach, the secretion of intrinsic factor by gastric parietal cells, and the subsequent binding of the cobalamin–intrinsic factor complex to specific receptors in the distal ileum for internalization. Notably, only 1% of vitamin B12 intake is absorbed via passive diffusion [5]. Following absorption, cobalamin binds to a specific transporter protein known as transcobalamin. This protein exists in three isoforms, with only transcobalamin-II serving as the biologically active form. Subsequently, cobalamin is distributed throughout the body or stored in the liver, where it can meet the body’s needs for some years [5]. There are only two reactions known in which vitamin B12 acts as a co-factor in the human body. The first is the conversion of homocysteine in methionine, a reaction occurring in the presence of folic acid derivate, which is fundamental to DNA synthesis. The second one is the conversion of methylmalonil-CoA into succinyl-CoA, a step for fatty acid synthesis required for myelin production [6]. Down-regulation of these two reactions during vitamin B12 deficiency causes the clinical and laboratory findings of cobalamin deficiency. Regarding the first reaction, vitamin B12 deficiency impairs DNA synthesis and induces dyssynchronous maturation of the nucleus and cytoplasm, resulting in altered cellular proliferation that manifests in tissues with high turnover; moreover, conversion of homocysteine is impaired. Consequently, typical findings are extravascular hemolysis with megaloblastic anemia, oral mucositis, and an increase in homocysteine blood concentration. In case of failure of the second reaction, vitamin B12 deficiency entails central and peripheral neuron demyelination with accumulation of methylmalonic acid in blood and urine [5].

Patients at high risk of vitamin B12 deficiency are those with low cobalamin intake, such as vegetarians or vegans, or those with conditions that impair its absorption. Among the latter, the most known is pernicious anemia, an autoimmune disorder in which severe atrophic gastritis develops with a virtual absence of intrinsic factor secretion. Noteworthy, cobalamin absorption is also impaired by several drugs such as proton pump inhibitors (increased gastric pH), metformin (interference with the binding between intrinsic factor and its receptor), or colchicine. Moreover, every condition that affects intestinal mucosal integrity as celiac disease or Crohn’s disease, as well as surgery (e.g., partial or total gastrectomy, ileal resection, or enteric diversion), could affect cobalamin absorption [4].

Overall, the prevalence of vitamin B12 deficiency in Western countries is estimated to be about 6% in persons < 60 years and close to 20% in adults> 60 years old and rises to peaks of more than 80% in vegans [4,7].

The clinical manifestations of vitamin B12 deficiency vary in severity depending on the degree and duration of the deficit, with instances where it may be asymptomatic and diagnosed incidentally [4,8]. Typical manifestations of mild deficiency include fatigue and anemia without neurological symptoms, while moderate deficiency may present with macrocytic anemia, glossitis, and mild neurological deficits. Severe deficiency can lead to bone marrow suppression with pancytopenia and pronounced neurological deficits [4]. However, up to 27% of patients with neurological signs do not have anemia [9] and neurological manifestations may be the earliest and only manifestation of cobalamin deficiency [10], even without macrocytosis [11]. Noteworthy, neurologic symptoms of cobalamin deficiency can mimic FM and range from the presence of neuropathic pain, paresthesia, poor coordination, ataxia, weakness, stiffness, sleepiness, or fatigue to neuropsychiatric symptoms such as depression, delusions, psychosis, or cognitive impairment [4,5,8,12]. Features of polyneuropathy associated with cobalamin deficiency include sudden onset of symptoms, symptoms onset in hands, and concomitant involvement of upper and lower extremities. Although the prevalence of criteria-defined FM in patients with vitamin B12 deficiency has not been elucidated, observational studies suggest that its supplementation may improve the severity of FM symptoms [13].

Vitamin B12 deficiency should be suspected in patients with FM with predominant neurological symptoms, especially if anemia and/or macrocytosis (mean corpuscular volume, MCV > 100 fL) are present, or in case of concomitant risk factors such as vegetarian/vegan diet or gastrointestinal disorders potentially leading to cobalamin deficiency [4,14]. Demonstration of low serum vitamin B12 level can confirm the diagnosis, but available assays have limited diagnostic performance and there are no consensus cutoffs to define deficiency [8]. Increased serum homocysteine and urine methylmalonic acid levels support diagnosis in patients with clinical suspicion and borderline or normal serum vitamin B12 levels [14].

Management of cobalamin deficiency aims to correct the cause of the deficiency, if possible, and perform vitamin replenishment [4]. In general, for cases with more serious symptoms and neurologic involvement, cyanocobalamin 1000 mcg/day intramuscularly or subcutaneously for 1–5 days and then 1000–2000 mcg/day orally is suggested while, for less severe conditions without neurologic symptoms, 1000 mcg/day orally is usually enough. Adequate treatment response is the normalization of homocysteine or methylmalonic acid levels within 1 week and normalization of MCV within 8 weeks [14].

**Summary**: Suspicion of vitamin B12 deficiency should arise in patients presenting with FM symptoms such as pain, fatigue, sleepiness, paresthesia, generalized pain, or sensory disturbances, especially in the presence of macrocytosis, glossitis, a vegan diet, gastrointestinal illness, or medications that could interfere with absorption. Diagnosis can be confirmed by low plasma cobalamin and high homocysteine levels.

### 2.2. Vitamin D Deficiency

Vitamin D is a fat-soluble vitamin primarily known for its role in bone metabolism and calcium–phosphorus balance. Vitamin D is partly taken from food, such as fatty fish or eggs, and mostly produced by the body at the skin level through exposure to sunlight [15]. Vitamin D deficiency is a highly prevalent condition, affecting nearly 24% of the general population [16] and reaching a peak of 78% in elderly individuals with fracture [17]. Risk factors for vitamin D deficiency are low exposure to sunlight, obesity, dark skin pigmentation, or every condition with impaired fat absorption as bariatric procedures, inflammatory bowel disease, biliary disorders, or pancreatic exocrine insufficiency [15]. Vitamin D is a pro-hormone that requires two consecutive hydroxylations by the liver and kidney into the biologically active form, 1,25-dihydroxyvitamin D, that acts by influencing several transcription factors in the nucleus of tissue targets [18]. The main effects of vitamin D are rising circulating calcium levels, by increasing gut and renal uptake and stimulating renal tubular excretion of phosphates, thus facilitating bone formation [15]. However, vitamin D is involved in the regulation of the expression of over 900 gene variants [19] influencing muscle, nervous, immune, endocrine, and cardiovascular systems, as well as hormone production [20,21]. Of note, vitamin D has shown neuroprotective effects and modulation of the sensitivity of nerve fibers to excitation mediated by pain stimuli, both at a central and peripheral level [22].

Clinical presentation of vitamin D deficiency depends on the severity of the deficiency. While mild deficiency is often asymptomatic, more severe deficiency can be responsible for non-specific widespread musculoskeletal pain, deep bone pain, or proximal muscle weakness [23]. To the best of our knowledge, no studies have investigated the prevalence of fulfilling FM criteria in unselected patients stratified by vitamin D status. However, a high prevalence of vitamin D deficiency (40–70%) has been reported in patients with FM and a correlation between vitamin D levels and measures of FM severity is likely to exist [24,25].

There is no consensus cut-off for vitamin D deficiency but can range from serum 25-hydroxyvitamin D < 10 ng/mL (25 nmol/L) to 20 ng/mL (50 nmol/L), while vitamin D insufficiency is commonly defined as serum 25-hydroxyvitamin D 21–29 ng/mL (52.5–72.5 nmol/L) [15]. Symptomatic patients with vitamin D deficiency may require high doses of vitamin D supplementation until adequate serum levels are achieved, followed by a maintenance dose to prevent recurrence; for example, 50,000 units per week of oral vitamin D supplement for 8 weeks to achieve serum 25-hydroxyvitamin D > 30 ng/mL and 1500–2000 units per day for maintenance [15].

**Summary**: In FM patients experiencing widespread musculoskeletal/bone pain and muscle weakness, consideration should be given to the possibility of vitamin D deficiency. Evaluation of serum 25-hydroxyvitamin D level is recommended.

### 2.3. Iron Deficiency

Iron is an essential trace mineral present in all cells and all tissues and is necessary for hemoglobin production and cellular metabolism, including DNA synthesis, mitochondrial energy generation, and catalysis of metabolic redox reactions [26]. The main risk factors for iron deficiency are low iron intake (e.g., vegetarian diet), impaired absorption (e.g., after bariatric surgery, celiac disease, H. pylori infection or drugs as antacids), or chronic bleeding (e.g., heavy menstrual bleeding, occult gastrointestinal blood loss, or multiple pregnancies) [27].

Iron deficiency, independently from anemia, causes functional impairment of skeletal muscle [28] potentially leading to symptoms commonly reported in FM such as fatigue, weakness, exercise intolerance, dyspnea, or restless leg syndrome [26]. Observational data suggest a higher prevalence of FM in iron deficiency anemia compared with healthy individuals (18% vs. 6%) [29]; furthermore, treatment with ferric carboxymaltose has been suggested to reduce pain levels in FM patients with low ferritin [30]. Of note, iron is a cofactor for several enzymes involved in the synthesis of neurotransmitters crucial to pain control, such as tryptophan hydroxylase (for serotonin) and tyrosine hydroxylase (for norepinephrine and dopamine) [31,32].

Other potential signs are dry and frail hair, koilonychia (nail deformity with longitudinal and/or transverse concave nail dystrophy with nail plate depressed centrally and everted laterally), atrophic glossitis and angular cheilosis (grayish-white thickening with adjacent erythema at corners of the mouth), and pica (compulsive eating of non-nutritive substances).

Diagnosis of iron deficiency is generally based on serum ferritin levels below 15–100 mcg/L or transferrin saturation (TSAT) below 16–20% (differential cutoffs in inflammation or other medical conditions, such as heart failure, are used) [26]. If iron deficiency is long-lasting, microcytic anemia (MCV < 80 fL) develops, unless other concomitant factors increasing MCV are present, such as folate or vitamin B12 deficiency and myelodysplasia. When iron deficiency anemia is found in adults > 50 years old without evident etiology, bidirectional endoscopy is recommended to rule out cancer [33]. First-line treatment of iron deficiency is, if possible, treating the cause and engaging in oral iron supplementation for at least 3 to 6 months. If patients are intolerant to different formulations of oral iron replacement therapy or if a quick recovery is needed, parental iron supplementation must be considered [34].

**Summary**: Iron status, including ferritin and TSAT, should be evaluated in patients with suspected FM and typical symptoms of iron deficiency (fatigue, weakness, exercise intolerance, dyspnea, restless leg syndrome, frail hair, koilonychia, atrophic glossitis, angular cheilosis, or pica) and/or microcytosis.

### 2.4. Magnesium Deficiency

Magnesium is a fundamental co-factor in many biochemical reactions in the human body [35]. Of note, magnesium is complexed with phosphate nucleotides (i.e., ATP, ADP, and GTP), and virtually, all reactions involving ATP require the presence of magnesium ions [36]. Moreover, magnesium has an important role in the synthesis of nucleic acids and proteins [37], in modulating cell proliferation, differentiation, and apoptosis [38], and is involved in ion transport by pumps, carriers, and ion channels [39]. Furthermore, magnesium is involved in pain pathways by influencing central neural sensitization by blocking N-methyl-D-aspartate (NMDA) receptors [40]. Magnesium homeostasis depends on the balance between intestinal absorption and renal excretion, with the rate of intestinal absorption and renal sparing inversely correlated to magnesium body storage through a mechanism finely controlled by different hormones, such as vitamin D, parathyroid hormone, and estrogen [41]. Magnesium is a prosthetic ion in chlorophyll; the main dietary sources of this ion are vegetables such as whole seeds, unground grains, legumes and green leafy vegetables, cocoa, nuts, and almonds [42]. Despite its widespread distribution, the daily intake of magnesium in most industrialized countries is insufficient and does not meet the current recommended daily allowance [43]. Hypomagnesaemia is defined as serum magnesium concentration <0.75 mmol/L, and it is frequently associated with other electrolyte abnormalities such as hypokalemia and hypocalcemia [41]. Magnesium deficiency is favored by some clinical conditions such as alcoholism, diabetes, intestinal malabsorption (i.e., inflammatory bowel disease or celiac disease), endocrinological disorders (i.e., thyroid dysfunction or hyperparathyroidism), chronic kidney disease, and use of drugs (especially proton pump inhibitors) [41]. Large quantities of magnesium are specifically needed by cells in organs with high metabolism, like the brain, heart, and muscles, and participate in muscle tone (including smooth muscle) and myocardial excitability.

Consequently, magnesium deficiency often manifests with an FM-like clinical picture including pain, fatigue, weakness, muscle cramps, impaired concentration and attention, sleep disturbances, anxiety, restlessness, palpitations/arrhythmia, and headaches [44,45]. Although the prevalence of FM in patients with magnesium deficiency is still not known and inconstant results have been obtained when investigating the relationship between magnesium level and FM symptoms, some evidence suggests that dietary intake of magnesium is lower in FM patients [46] and that supplementation may be beneficial [47]. Thus, in case of deficiency, treatment is based on supplementation, using magnesium inorganic or organic (e.g., citrate, gluconate, aspartate) salts with the latter showing higher bioavailability [48].

**Summary**: In FM patients presenting with predominant muscular and cognitive symptoms, consideration should be given to the possibility of magnesium deficiency.

### 2.5. Vitamin C Deficiency

Vitamin C, also known as ascorbic acid, is a hydrophilic vitamin whose main known function is being an essential cofactor for hydroxylation of proline to hydroxyproline in collagen synthesis and hydroxylation of neurotransmitter dopamine to noradrenaline [49]. Humans cannot synthesize or store vitamin C; therefore, adequate and regular intake of vitamin C is crucial for its function. The main sources of vitamin C are fresh and raw vegetables and citrus fruits because heat and storage degrade ascorbic acid.

Early symptoms of vitamin C deficiency can resemble FM and include fatigue, malaise, lethargy, mood changes, myalgia, or bone pain; the classic full picture (scurvy) manifests later with ease of bleeding (petechiae, perifollicular hemorrhages, easy bruising), teeth loosening, and anemia [49]. However, scurvy still remains rare in Western countries with adequate dietary availability [50] although the incidence is higher in alcoholism, low socioeconomic status, elderly with loneliness, some severe food allergies, extremely fat diets, or severe psychiatric illness [49,50]. Furthermore, a higher prevalence of vitamin C deficiency is reported in gut diseases inducing malabsorption or end-stage renal disease requiring dialysis [49]. Of note, some clinical evidence supports a relationship between pain and vitamin C deficiency [51]. In addition to the well-known high prevalence (80%) of musculoskeletal pain among the classic symptoms of scurvy, often due to micro-bleeds in soft tissues or joints [52], vitamin C has been associated with conditions characterized by dysfunctional processing of pain, such as complex regional pain syndrome (CRPS). Indeed, observational studies indicate that daily administration of vitamin C can decrease the incidence of CRPS following fracture or surgery [53].

Vitamin C deficiency must be suspected in the presence of typical clinical manifestation in subjects with high risk for dietary insufficiency and could be considered confirmed if clinical manifestations resolve with vitamin C supplementation [50]. If the serum dosage of vitamin C is available, the normal range should be considered between 0.2 and 1.9 mg/dL (11–108 mcmol/L). Vitamin C supplementation therapy consists of 500–1000 mg/day for 1 month or until full recovery [50].

**Summary**: Vitamin C deficiency should be suspected in FM patients with risk factors such as alcoholism, advanced age, low socioeconomic status, extreme dietary habits, or severe gastrointestinal or renal conditions.

## 3. Endocrine Diseases

### 3.1. Thyroid Diseases

Considering the critical role of thyroid hormones in regulating essential metabolic processes, including growth, protein synthesis, and sensitivity to catecholamines, it is inherent that both deficiency and excess can lead to complex symptomatology resembling FM.

Hypothyroidism is a condition characterized by thyroid hormone deficiency [54]. It is common worldwide with a reported prevalence of 0.2–5.3% in Europe and up to 7% in the elderly [55]. Risk factors for hypothyroidism are female sex, older age, and family history of thyroid or autoimmune diseases. Primary hypothyroidism is the most common form, and it is due to thyroid gland failure. Autoimmune thyroiditis (Hashimoto’s thyroiditis, HT) is the most frequent cause of thyroid dysfunction in iodine-sufficient populations while less common etiologies are severe iodine deficiency, iatrogenic injury of thyroid as radioiodine therapy for hyperthyroidism, radiation therapy for head or neck cancer and thyroid surgery, or use of certain drugs including amiodarone, lithium, metoclopramide, tyrosine kinase inhibitors, or interferon-alfa [54]. Central hypothyroidism includes forms of secondary hypothyroidism, due to pituitary failure with thyroid-stimulating hormone (TSH) deficiency, or tertiary hypothyroidism due to hypothalamic failure with thyrotropin-releasing hormone (TRH) deficiency. Most cases of central hypothyroidism are due to a compressive lesion of the pituitary sella, such as pituitary adenomas or meningiomas, cranial surgery or radiation therapy, autoimmune disease (hypophysitis) [56] or drugs such as glucocorticoids [54]. Peripheral hypothyroidism can be caused by inappropriate expression of the deiodinase-3 enzyme, which inactivates thyroid hormone, or rare genetic syndromes that lead to reduced sensitivity to thyroid hormone [54].

Typical symptoms of hypothyroidism are fatigue, muscle cramps and weakness, arthralgia, cold sensitivity, paresthesia, impaired mood, slow mental activity, lethargy, weight gain, dry skin, hair loss, and menstrual irregularities; in severe forms, patients may present with altered mental status, hyponatremia, bradycardia, and progressive lethargy with multiple organ dysfunction. However, hypothyroidism may be frequently asymptomatic, especially subclinical hypothyroidism, or characterized by nonspecific symptoms in particular in older adults [54,57].

Hyperthyroidism is a clinical state characterized by excessive thyroid hormone levels resulting from inappropriately high synthesis and secretion of thyroid hormone [58]. The prevalence of hyperthyroidism is estimated at about 1.2% in the general population, with a higher peak in females and iodine-deficient regions [58,59]. In most cases, hyperthyroidism is due to an increase in thyroid gland hormone production, such as Graves’ disease, toxic thyroid adenoma, or toxic multinodular goiter. However, there are other less frequent etiologies such as TSH-producing pituitary adenoma, functional thyroid cancer metastases, thyroid hormone resistance, or drugs-induced iodine overload (i.e., amiodarone, iodinated contrast media).

Graves’ disease is the most common cause of hyperthyroidism. This is an autoimmune disease characterized by the development of autoantibodies that bind to and activate the TSH receptor present on thyroid follicular cells, causing hyperthyroidism, and possibly on subsets of cells within the orbit and skin, inducing orbitopathy [60]. The exact pathogenesis of Graves orbitopathy is incompletely understood. However, periorbital fibrocytes infiltrate with local edema, and fibroblastic activation was seen as a possible cause of exophthalmos, corneal xerosis, and sometimes diplopia [61,62]. Toxic thyroid adenoma is a single follicular thyroid nodule that produces excess thyroid hormones, resulting in hyperthyroidism, while toxic multinodular goiter is an enlarged thyroid gland with multiple autonomously functioning nodules [58].

Hyperthyroidism can present with varied and non-specific symptomatology, including fatigue, nervousness, anxiety, poor concentration, insomnia, tremor, muscle weakness, heat intolerance, increased sweating, palpitations, involuntary weight loss, and, in the case of Graves’ disease, eye symptoms such as eye irritation, pain, discomfort in eye motion, and diplopia [63].

The bidirectional association between FM and thyroid diseases, HT, in particular, has been largely studied [64]; a prevalence of up to 70% of thyroid autoimmunity has been reported in FM patients. On the other hand, up to 40% of patients with HT satisfy the criteria for FM [65].

Diagnosis of both hypo- and hyperthyroidism relies on the evaluation of thyroid function tests. In all patients with clinically suspected thyroid dysfunction, guidelines suggest obtaining TSH as an initial test and, if abnormal, assessing FT4 [58,66].

The finding of an elevated TSH and low FT4 indicates primary hypothyroidism due to disease in the thyroid gland; a low TSH and low FT4 indicates hypothyroidism due to a problem involving the pituitary gland. A low TSH with an elevated FT4 is found in individuals who have hyperthyroidism. Further testing, e.g., thyroid-specific autoantibodies or imaging will be used to assess the specific cause.

**Summary**: Thyroid dysfunction may manifest with nonspecific symptoms resembling those of FM, such as fatigue, weight loss/increase, tremor, anxiety, heat/cold intolerance, palpitations, or insomnia. Evaluation of TSH levels is recommended, and if abnormal, further assessment of FT3 and FT4 is suggested.

### 3.2. Obesity

Obesity is a multifactorial disease characterized by an increase in the percentage of body fat due to a positive energy imbalance, with increased energy intake and/or decreased energy expenditure. There are several factors involved in this dysfunctional energy homeostasis, such as behavioral, sociocultural, environmental, genetic, epigenetic, and physiological aspects [67]. The most simple and efficient tool to classify obesity is body mass index (BMI), defying obesity with a BMI > 30 kg/m^2^. Obesity prevalence worldwide is 10.8% in males and 14.9% in females [68], with peaks of 37% and 41%, in males and females, respectively, in some Western countries such as the United States [69].

Obesity is a key feature of metabolic syndrome, a condition characterized by chronic inflammation and insulin resistance, increasing the risk of developing type 2 diabetes, non-alcoholic fatty liver disease, and cancer [66]. Moreover, obesity is associated with pulmonary illness as restrictive disorders or obstructive sleep apnea, musculoskeletal disorders such as osteoarthritis or low-back pain, and psychiatric disorders [66]. This condition is associated with a high prevalence of depressive symptoms, anxiety, and perceived stress [70]. Overall, obesity is associated with an increased risk of all-cause mortality [71] and reduced quality of life [72].

Obesity is not only a risk factor, but a disease itself. Commonly reported symptoms of obesity are weakness, shortness of breath, and musculoskeletal pain. Moreover, a rare obesity-associated condition is Dercum disease, also known as *adiposis dolorosa*. This is characterized by chronically and severe painful subcutaneous tissue of unknown etiology, with frequently observed painful lipomas in the buttocks, upper arms, thighs, and abdomen. There are no specific laboratory or imaging findings, and management is not standardized and has limited proof of efficacy [73].

A bidirectional relationship between obesity and FM is already well-known in the literature [74] with 30–40% of obese patients satisfying the criteria for FM and approximately the same percentage of FM patients being obese. Obesity is associated with different domains of the disorder, including composite measures of activity, pain severity, tender point count, stiffness, fatigue, physical functioning/disability, sleep, cognitive dysfunction, and quality of life [75]. Furthermore, clinical studies addressed the effect of therapeutic weight loss in FM, either by bariatric surgery, diet/exercise combination, or behavioral weight loss, providing preliminary evidence for a potential benefit of weight loss in ameliorating FM symptoms [75,76].

The primary approach to treating obesity involves lifestyle interventions focused on achieving a 5% to 10% reduction in body weight within 6 months. This typically entails adopting a low-calorie diet, resulting in a daily energy deficit of 500–750 kcal, alongside implementing an exercise regimen comprising at least 150 min per week of moderate-intensity aerobic activity for most patients. Should lifestyle interventions prove ineffective, approved medications or bariatric surgery have shown efficacy in addressing body weight and associated obesity-related conditions [66].

**Summary**: There is a well-known association between obesity and FM; subjects with obesity could present weakness, sleep disturbances, musculoskeletal pain, and mood disturbances. In those patients, weight loss should be prioritized, not only to improve global health and reduce the incidence of obesity-associated chronic diseases but also to alleviate FM symptoms.

## 4. Cardiopulmonary Diseases

### Obstructive Sleep Apnea

Cardiorespiratory fitness (CRF) refers to the capacity of the cardiovascular and respiratory systems to supply oxygen to muscles, and other bodily tissues, during exertion, and it is intuitive that patients with a wide array of cardiovascular and respiratory diseases may experience musculoskeletal symptoms resembling FM.

Obstructive sleep apnea (OSA) is a sleep disorder characterized by repetitive complete (apnea) or partial (hypopnea) upper airway obstruction during sleep leading to recurrent arousals and sleep fragmentation [77]. OSA, defined as an apnea–hypopnea index (AHI) exceeding 5 events per hour, is a prevalent condition in Western countries, affecting approximately 10% of adult females and 20% of adult males [78]. Major risk factors for OSA are aging, obesity (in particular BMI over 35 kg/m^2^), heart failure, acromegaly, hypothyroidism, smoking, and craniofacial abnormalities such as retrognathia or anatomical alterations causing obstructions to airflow at the nasal level [77,79]. All these conditions reduce the caliber of upper airways, facilitating their collapse during the inspiratory sleep phase, a moment in which pharyngeal dilator muscles have a reduced tone, leading to airflow impairment [80].

Typical symptoms of OSA are sleep problems, such as restless and non-refreshing sleep, snoring, awakening with paroxysmal nocturnal dyspnea, and sleep fragmentation; moreover, patients refer excessive daytime sleepiness with decreased concentration and memory, morning headaches, and sometimes nocturia. However, patients could have only fatigue as an OSA symptom [77,81].

Notably, sleep disturbances, cognitive fog, and fatigue are recognized as key symptoms of FM, highlighting the importance of considering OSA in the differential diagnosis. To emphasize this need, some studies suggested a bidirectional association between OSA and FM with up to 85% of patients referred to a sleep clinic having a concurrent diagnosis of FM [82,83].

In the presence of symptoms, polysomnography is the gold standard diagnostic test for OSA and for excluding other non-obstructive causes of sleep disturbance [84]. OSA management includes lifestyle intervention for weight loss, positional therapy to keep the patient in a non-supine position, oral appliances (such as nasal dilators or mandibular advancement devices), and use of continuous positive airway pressure (CPAP); in selected subjects who fail or do not tolerate first-line therapies, surgery may be indicated [85]. Overall, OSA treatment with CPAP was demonstrated to reduce daytime sleepiness and fatigue and improve self-perceived energy and quality of life in adults with confirmed OSA [86,87].

**Summary**: Individuals with obstructive sleep apnea (OSA) may exhibit typical FM symptoms such as fatigue, sleep disturbances, and decreased concentration and memory. High suspicion for OSA arises when daytime sleepiness is reported or in the presence of predisposing factors such as obesity or anatomical abnormalities causing narrowing of the upper airways.

## 5. Neurological Diseases

Chronic pain commonly accompanies numerous neurological disorders, impacting 20–40% of patients across various primary neurological conditions. These disorders stem from diverse mechanisms, such as traumatic injuries to the central nervous system (CNS), neurodegeneration, and neuroinflammation [88].

The prototypical neurological disease associated with pain is peripheral neuropathy, a term encompassing a diverse array of conditions marked by nerve damage occurring outside the CNS. The prevalence of peripheral neuropathy in the general population is about 2.4–3% [89], and its etiology often is idiopathic [90]. Neuropathy may arise from damage to axons, myelin sheath, nerve cell body, connective tissue, blood vessels, or a combination of these factors. The leading causes of peripheral neuropathy are widely recognized as diabetes and excessive alcohol consumption. Additionally, contributing factors may include malnutrition, particularly deficiencies in vitamins B12, B1, or B6, as well as certain medications like chemotherapy agents, amiodarone, colchicine, and gold salts. Moreover, conditions such as monoclonal gammopathies, including monoclonal gammopathy of undetermined significance, multiple myeloma, light chain amyloidosis, and Waldenstrom macroglobulinemia, along with heavy metal intoxications, can also play a significant role. Symptoms of neuropathy can range from pain (commonly described with neuropathic qualifiers such as burning, shooting, pricking, pins and needles, squeezing, or freezing pain) often associated with allodynia and hyperalgesia, paresthesia, weakness to imbalance, and/or autonomic dysfunction, anatomically consistent with the distribution of the specific peripheral nerve affected. Neuropathies could be classified based on the extent of nervous involvement (i.e., mononeuropathies, multiple mononeuropathies, distal symmetric polyneuropathy, small fiber neuropathy, polyradiculoneuropathies, plexopathy) or based on the predominant impairment of sensory, motor, or autonomic functions [91,92]. In subjects with sensory, autonomic, or motor disturbance, electrodiagnostic tests, such as nerve conduction studies or needle electromyography, may confirm the diagnosis in most cases and give information on fiber type or affected region of nerve fiber involved [93].

However, in the clinical setting of generalized musculoskeletal pain, the primary mimicker of FM is small fiber neuropathy (SFN), a disorder of the small myelinated Aδ-fibers and unmyelinated C-fibers [94]. Symptoms of SFN are highly overlapping with those of FM and include fatigue, cognitive disturbances, widespread musculoskeletal pain, and a large array of manifestations attributable to autonomic dysfunction (e.g., sicca syndrome, accommodation problems, orthostatic complaints, hypohidrosis or hyperhidrosis, hot flashes, intestinal problems, gastroparesis, impotence, decreased ejaculation or lubrication, and palpitations). Unfortunately, SFN cannot be captured by standard nerve conduction studies, since these only reflect the function of larger, myelinated fibers. Other tests, such as skin biopsy to quantify intraepidermal nerve fibers, are now recognized as sensitive and specific tools to assist the diagnosis of SFN that anyway suffers limited availability and inadequate standardization of techniques. The need for considering SFN in the setting of suspected FM is emphasized by the fact that 50% of patients with FM have evidence of SFN as underlined in a recent meta-analysis [95]. Thus, differentiating primary FM from SFN is challenging, and currently, there is no clear evidence of which FM patient should be screened for suspected SFN [96].

**Summary**: Patients experiencing widespread pain should be evaluated for peripheral neuropathy, with small fiber neuropathy (SFN) being of particular concern. SFN should be considered in FM patients, especially when neuropathic symptoms such as paresthesia, autonomic disturbances, sweating abnormalities, and hypoesthesia to warm or cold stimuli, along with skin changes, are present.

## 6. Infections

Pain serves as a prominent signal of acute infection. Furthermore, post-infection, inflammation may trigger an unusual immune response, resulting in various types of pain, be it acute or chronic, neuropathic, nociceptive, or nociplastic. Alternatively, it can function as a psychological stressor, instigating or intensifying chronic pain [97,98,99]. Postherpetic neuralgia, characterized by persistent pain lasting beyond 90 days following the emergence or resolution of the distinctive vesicular rash caused by the varicella-zoster virus (VZV), represents the prototypical infectious pain syndrome, affecting roughly half of the infected individuals (105). Additionally, other members of the Herpesviridae family have been linked to painful syndromes, typically localized but occasionally manifesting as widespread pain on one side of the body [100]. In most cases, these painful syndromes do not fall within the differential diagnosis of FM, as the pain is not truly generalized and lacks the accompanying symptoms of FM (e.g., fatigue or sleep disturbances). Additionally, the presence of mucocutaneous manifestations is usually observed.

However, other chronic infections may present with musculoskeletal pain as part of their clinical spectrum, underscoring the importance of considering these conditions in the differential diagnosis.

The prognosis for human immunodeficiency virus (HIV) infection has significantly improved with the availability of anti-retroviral treatments, leading to a near-normal lifespan for HIV patients, although these treatments rarely eradicate HIV infection. Chronic pain affects over 50% of individuals living with HIV, with distal peripheral neuropathy being the most prevalent cause. However, there is also an increased risk of experiencing concurrent nociceptive or nociplastic pain [101,102]. Indeed, HIV patients frequently report symptoms such as abdominal pain, chest pain, musculoskeletal pain, headaches, and fatigue, which can be diagnosed as FM in nearly 40% of patients [101].

Similarly, hepatitis C virus (HCV) infection is estimated to affect 71 million people worldwide [103]. Beyond the liver involvement, HCV-infected subjects could have extra-hepatic manifestations such as arthralgia, frank arthritis, or peripheral neuropathy with painful paresthesia or satisfy criteria for FM in 25% of cases [104,105].

More recently, severe acute respiratory syndrome coronavirus 2 (SARS-CoV-2) has emerged as a virus capable not only of inducing complex clinical presentations during acute infection but also of leaving persistent sequelae, defined as long COVID-19 or post-COVID-19 conditions (PCCs).

A recent meta-analysis underlined the persistence of symptoms in 30% of patients two years after COVID-19 [106], in particular, fatigue (28%), cognitive impairment (28%), sleep problems (21%), and pain (8%). These symptoms have a large degree of overlap with FM; in an early report from our group, 30% of patients satisfied the classification criteria for FM after SARS-CoV-2 infection [107]. Furthermore, a large array of rheumatic manifestations have been reported during all the phases of the disease [108,109,110,111].

Finally, the bacterium *Borrelia burgdoferi* causes a tick-borne illness called Lyme disease, the most common vector-borne infectious disease in the temperate northern hemisphere [112]. Borrelia species are carried in the midgut of ticks, particularly Ixodes species. When an infected tick takes a blood meal, Borrelias multiply, undergo phenotypic changes, and, once injected, spread hematogenously [113]. The early phase of Lyme disease manifests as localized erythema migrans, a single skin lesion at the site of the tick bite that occurs in 70–80% of infected persons and begins 3–30 days after the bite. Erythema migrans appears as an area of round, flat, or slightly raised, erythema that usually has an area of central clearing configuring a classic target lesion (but appearance can vary widely) and could be associated with systemic symptoms such as fever, headache, malaise, fatigue, and arthralgias [113]. After a few days, signs and symptoms of early infection spontaneously resolve. Weeks or months after bites, a second phase of Lyme disease could manifest as cardiac involvement (myocarditis or atrioventricular block) and/or frank arthritis, which could be monoarticular or oligoarticular, usually involving the knee with intermitting trend [112]. If untreated, chronic Lyme manifests its late phase (days to months after tick bite) with neurological symptoms such as encephalomyelitis, peripheral neuropathy or encephalopathy, arthritis, or less specific symptoms such as chronic or intermittent musculoskeletal pain, heart palpitations or an irregular heartbeat, dizziness, fatigue, or cognitive impairment [113]. The complexity of clinical presentation can make the diagnosis of Lyme disease challenging, especially when tick bite or initial cutaneous manifestations are not recognized during early stages, often resulting in misdiagnoses, including FM. In a prospective study of 287 patients treated for confirmed Lyme disease, 8% developed FM within 5 months of treatment [114].

Diagnosis is confirmed if two-tiered serologic tests (typically an enzyme immunoassay and an immunoblot) for Borrelia antibodies are both positive. If high clinical suspicious and initial laboratory tests are negative, repeating tests after 2–3 weeks is suggested to increase sensitivity [113]. First-line therapy for Lyme disease is doxycycline 200 mg orally per day. Treatment duration varies between disease phases, from 10 days in the cutaneous phase, 14–21 days in cardiac or neurologic involvement, and 28 days in arthritis [112].

Of note, although patient-reported symptoms, typical of FM, such as fatigue, cognitive dysfunction, and musculoskeletal pain, are reported in all phases of untreated Lyme disease; the trickiest setting is the persistence of symptoms in a subgroup of individuals who had received treatment. The term post-treatment Lyme disease syndrome (PTLDS) was coined to capture symptoms (fatigue, widespread musculoskeletal pain, and/or cognitive impairment) persisting for longer than 6 months post-treatment [15]. These subjective symptoms must be continuous or relapsing for at least 6 months following completion of treatment and must be severe enough to reduce functional ability in the patient’s life.

**Summary**: Chronic infections can manifest with musculoskeletal symptoms resembling FM. Testing for HIV and HCV should be considered in sexually active individuals on intravenous drug users, while a recent history of COVID-19 may indicate long COVID-19. Additionally, testing for Lyme disease may be warranted in patients potentially exposed to tick bites, especially in endemic areas.

## 7. Cancer

Cancer is the second cause of death worldwide [115]. Pain and fatigue are frequent but non-specific signs of neoplastic diseases [116,117], and some evidence suggests that widespread musculoskeletal pain is associated with cancer mortality [118]. In fact, pain and fatigue are not only common in various types of advanced-stage cancer, but they are also frequently reported as the initial symptoms in newly diagnosed stage IV cancer patients, with 61% experiencing back pain and 47% experiencing fatigue [119]. Cancer pain depends on several etiologies such as nociceptive (e.g., bone metastasis or other tissue injuries), neuropathic (e.g., compression/invasion- or drug-induced peripheral neuropathy), or stress-related (e.g., anxiety, depression, and hopelessness) [120].

Bone metastases represent a common complication of cancer, with a remarkably high incidence in multiple myeloma (70–95%) [121], prostate cancer (65–90%), breast cancer (65–75%), and lung cancer (17–64%) [122]. The incidence of bone metastasis as the initial manifestation of cancer ranges from 10% to 23% of cases where metastasis is the first sign of the disease, which accounts for 3% to 4% of all cancer cases [123]. Bone metastasis is the most frequent cause of cancer-induced pain [124]. Cancer bone pain ranges from dull, vague, and persistent pain to intermittent, sharp, and severe pain, often worsening with physical activity, that involves mainly vertebrae, pelvis, femur, ribs, and skull [116]. Subjects with bone metastasis often experience complications such as pathological fractures, spinal cord compression with impaired mobility, and hypercalcemia [125] that can further contribute to pain, and in many cases, the complication may precede the diagnosis of cancer [126,127]. The potential occurrence of multiple bone metastasis in patients with unrecognized primary malignancy can potentially lead to multifocal pain resembling FM; in such cases, when the clinical suspicion of malignancy is high, panoramic imaging such as whole-body MRI or PET/CT scan may be requested for accurate diagnosis [128].

In addition to bone metastasis, muscle dysfunction is a prevalent phenomenon in the oncology setting where patients across a wide range of diagnoses develop impaired muscle function and myalgia regardless of tumor stage and nutritional state [129]; in a small proportion of patients, muscle manifestation can be attributable to frank paraneoplastic inflammatory myopathy characterized by progressive muscle weakness, pain, creatine kinase (CK) elevation, and visceral manifestations such as dyspnea and dysphagia. Of note, myositis can precede the onset of cancer up to three years [130]. The risk of a diagnosis of cancer is highest during the first year after the diagnosis of myositis.

In summary, according to observational evidence, the prevalence of FM is increased in cancer patients [131]; thus, although not common in clinical practice, some patients evaluated for musculoskeletal symptoms suspected of FM may indeed have an unrecognized underlying neoplasm.

Unfortunately, there is no universal testing that can be carried out systemically to rule out cancer. Demographic data (age, gender), risk factors (smoke, exposure to carcinogens, familial history), specific organ involvement, presence of laboratory abnormalities, or worsening pain/fatigue with poor response to treatment, may warrant additional testing on a case-by-case basis.

**Summary:** In patients presenting with multifocal musculoskeletal pain along with worsening fatigue, constitutional symptoms, or organ-related manifestations, particularly in the presence of risk factors, the possibility of cancer should always be considered.

## 8. Celiac Disease

Celiac disease (CD) is a relatively common autoimmune disorder primarily affecting the small intestine and characterized by an aberrant immune reaction triggered by exposure to dietary gluten, a storage protein present in wheat, rye, barley, spelt, and kamut. The estimated prevalence of CD is 0.7–1.4%. Although the typical presentation is characterized by gastrointestinal symptoms such as diarrhea, constipation, nausea, vomiting, abdominal pain, abdominal distention, and weight loss, a significant proportion of patients can present an atypical presentation, which includes extra-intestinal symptoms like delayed puberty, amenorrhea, iron deficiency anemia, osteoporosis, elevated hepatic transaminase levels, as well as neurologic or psychiatric disorders [132]. Amongst extra-intestinal manifestations, classic symptoms of FM such as arthralgia, fatigue, myalgia but also anxiety, depression, and irritability are often reported [133]. A study published in 2013 suggested that 7% of patients with FM and gastrointestinal manifestations previously attributed to irritable bowel syndrome had unrecognized CD and responded well to a gluten-free diet [134].

The diagnostic approach to CD relies primarily on the detection of tissue transglutaminase IgA antibodies (tTG-IgA); in subjects with IgA deficiency, IgG against tTG, endomysial (EMA), or deamidated gliadin peptides (DGP) could be considered [135]. Diagnosis of CD is further confirmed by duodenal biopsy demonstrating villous atrophy [132]. Treatment of celiac disease is a lifelong gluten-free diet, which is effective in improving both classic [136] and extra-intestinal symptoms [137].

**Summary:** CD should be considered in patients exhibiting musculoskeletal symptoms suggestive of FM alongside concomitant gastrointestinal symptoms.

## 9. Drug-Induced Musculoskeletal Pain

Most medications produce several effects, but usually only one—the therapeutic effect—is wanted for the treatment of a specific disorder. The other effects may be regarded as unwanted, whether they are intrinsically harmful or not. These adverse drug reactions (ADRs) are extremely common [138] and can affect virtually all organs and systems in the body—including the musculoskeletal system—ranging from mild (e.g., muscle cramps) to life-threatening conditions (e.g., severe rhabdomyolysis) [139].

Many of these musculoskeletal ADRs do not pose significant challenges in terms of differential diagnosis with FM. This is because they are either linked to short-term medication use (e.g., quinolones) or because musculoskeletal symptoms are easily recognized as iatrogenic and lead to premature discontinuation of the causing agent. This paragraph will focus solely on the main classes of medications used for chronic or prolonged therapy that can result in long-lasting musculoskeletal symptoms, potentially leading to confusion with FM.

### 9.1. Lipid-Lowering Drugs

Statins (HMG-CoA reductase inhibitors) represent the cornerstone of lipid-lowering therapy and are amongst the most prescribed molecules worldwide for cardiovascular prevention. While generally well-tolerated and safe, statins have been associated with musculoskeletal ADRs ranging from mild myalgia to severe rhabdomyolysis and necrotizing myopathy [140]. Although considered a typical ADR of statins, data from clinical trials demonstrate that the excess of muscle pain due to statins is indeed low and >90% of all reports of muscle symptoms by participants allocated statin therapy were not clearly due to the statin [141].

While the onset of musculoskeletal pain typically occurs within 30–40 days following initial exposure, allowing for easy recognition of its iatrogenic nature, muscle pain can manifest even a year or more after treatment initiation [142]. In the latter case, some clinicians may be unfamiliar with statin ADRs and attribute symptoms to FM. However, a recent study suggested that administering statin therapy does not distinctly correlate with a worsened symptom burden in FM patients [143].

The pathophysiology behind muscular symptoms associated with statins remains incompletely understood, and currently, discontinuation of the drug represents the sole effective treatment. Moreover, employing the minimum effective dosage to attain the desired therapeutic effect stands out as the most efficacious approach for preventing muscular ADRs [144]. Other lipid-lowering agents, such as ezetimibe [145], fibrates [146], and the recently approved proprotein convertase subtilisin/kexin type 9 (PCSK9) inhibitors [147], have been all associated with musculoskeletal ADRs; in these cases as well, discontinuation of the drug is almost always associated with an improvement in pain symptoms [148].

### 9.2. Bisphosphonates

Bisphosphonates (BPs) are the most prescribed class of medication for the treatment of osteoporosis. Intravenous BPs, notably zoledronic acid, often (30%) cause an acute phase response resulting in an influenza-like syndrome such as intense musculoskeletal pain, fever, headache, malaise, and fatigue, sometimes accompanied by nausea, vomiting, and diarrhea [149]. Such acute ADR is most common following the first administration, begins approximately 24 to 72 h following the infusion, and resolves within 72 h. On the other hand, musculoskeletal pain can be less often associated with oral BP, in nearly 5% of cases [150]. While symptoms are typically mild, there have been reports of severe pain [151]. In these cases, the onset of musculoskeletal pain can occur anywhere from two days to two years after the initiation of oral BP treatment [150], complicating the association with the medication; in some cases, these symptoms may be erroneously attributed to FM.

### 9.3. Aromatase Inhibitors

Aromatase inhibitors (AIs) represent a cornerstone of therapy for estrogen-receptor-positive breast cancer in postmenopausal women [152].

Unfortunately, AI treatment leads to estrogen depletion, which in turn can lead to unpleasant side effects such as menopausal symptoms like flushing, insomnia, increased risk of ischemic heart disease, accelerated bone loss leading to higher osteoporosis risk, and most significantly, arthralgia. This ADR presents as joint pain affecting the hands, wrists, knees, lower back, hips, shoulders, and feet, with the fingers being the most affected. The mean time to symptom onset is 1.6 months after initiation of therapy, and symptom severity often peaks at 6 months [153].

The joint pain induced by AI therapy, or “aromatase-inhibitor-associated musculoskeletal syndrome” (AIMSS), is considered a leading cause of premature discontinuation; approximately 50% of patients will report new onset or worsening joint pain 1 year after therapy initiation [154].

### 9.4. Isotretinoin

Oral isotretinoin is a well-established treatment for severe acne and for acne that has not responded to oral antibiotics plus topical agents [155].

Although the exact pathogenesis is unclear, musculoskeletal pain and arthralgia are common rheumatological ADRs of the drug that can be detected in 20% of patients. Other uncommon musculoskeletal disorders related to isotretinoin are hyperostosis, extraspinal calcifications, enthesitis, arthritis, costochondritis, osteoporosis, growth retardation, premature epiphyseal closure in children, and as well as gout [156,157].

### 9.5. Others

A wide range of other molecules, belonging to numerous pharmacological classes, have been associated over the years with musculoskeletal ADRs, with highly variable incidence rates. Myalgias can also be caused by antihypertensive drugs such as ACE inhibitors, angiotensin-II receptor inhibitors, and calcium-channel blockers, as well as NSAIDs, proton pump inhibitors (PPIs), some oral antidiabetics, serotonin reuptake inhibitors (SSRIs), as well as antipsychotics and oral iron supplements [158]. In almost all cases, specific treatment is not required, except for discontinuation of the drug that caused the damage; obviously, early recognition of the underlying cause of these pathological conditions is crucial to avoid irreversible consequences.

**Summary:** Several drugs commonly used to treat chronic diseases have the potential to induce musculoskeletal symptoms resembling FM. A detailed drug history should be obtained in all patients, and a trial of treatment discontinuation, if feasible, may be warranted to establish a causative role of the medication.

## 10. Conclusions

In conclusion, several medical conditions commonly encountered in clinical practice may present with symptoms that overlap, at least in part, with those reported in FM, underscoring the critical need for accurate differential diagnosis. The range of potential mimickers is broad encompassing nutritional deficiencies, endocrine, metabolic, respiratory, gastrointestinal, neurological, infectious, and even neoplastic diseases. Despite the absence of comprehensive guidelines for differential diagnosis, heightened awareness among healthcare professionals regarding the potential mimickers of fibromyalgia is crucial to reducing instances of misdiagnosis. Consequently, patients will benefit from more appropriate and effective treatments, leading to better overall health outcomes. This approach underscores the importance of continued education and research in the field to enhance diagnostic precision and patient care.

## Figures and Tables

**Table 1:** Common non-rheumatic medical conditions mimicking fibromyalgia.

Medical Condition	Predisposing Factors	Typical Features	Clues to Diagnosis in FM Patients	First-Level Diagnostic Test
**Micronutrient deficiency**				
Vitamin B12 deficiency	Vegetarian or vegan diet, atrophic gastritis, drugs (PPIs, metformin, colchicine), inflammatory bowel disease, history of gastrointestinal surgery	Megaloblastic anemia, extravascular hemolysis, pancytopenia, neurological manifestations, glossitis	Chronic widespread pain and/or fatigue, with macrocytosis, or anemia/pancytopenia, glossitis, and prominent neurological manifestations (sleepiness, muscle weakness, paresthesia, ataxia)	↓ Vitamin B12 ↑ Homocysteine
Vitamin D deficiency	Low sunlight exposure, dark skin pigmentation, obesity, inflammatory bowel disease, chronic kidney disease, chronic liver disease, history of gastrointestinal surgery	Bone fragility	Chronic widespread pain/bone pain and/or fatigue with proximal muscle weakness	↓ 25-hydroxyvitamin D
Iron deficiency	Vegetarian or vegan diet, celiac disease, inflammatory bowel disease, chronic bleeding, history of gastrointestinal surgery, drugs (PPIs, antiacids)	Microcytic anemia, dry/frail hair, koilonychia, atrophic glossitis, angular cheilosis, restless leg syndrome, pica	Chronic fatigue/exercise intolerance with microcytosis or restless leg syndrome, frail hair, koilonychia, atrophic glossitis, and angular cheilosis or pica	↓ Ferritin ↓ TSAT
Magnesium deficiency	Alcoholism, diabetes mellitus, inflammatory bowel disease, chronic kidney disease, thyroid/parathyroid disorders), drugs (PPIs)	Neuromuscular hyperexcitability, cardiac arrhythmias, hypokalemia, hypocalcemia	Chronic widespread pain and/or fatigue with prominent muscular (e.g., cramps) and cognitive (agitation, restlessness) symptoms or palpitations/arrhythmia	↓ Magnesium
Vitamin C	Alcoholism, insufficient dietary intake (raw vegetables and citrus fruits), chronic kidney disease, inflammatory bowel disease	Scurvy	Chronic widespread pain and/or fatigue with easy bruising and bleeding or teeth loosening	↓ Vitamin C
**Endocrine disorders**				
Thyroid diseases	Female sex, family history of thyroid or autoimmune diseases, iodine deficiency, radioiodine therapy, neck radiation, drugs (amiodarone, lithium, metoclopramide, tyrosine kinase inhibitors, interferon-alfa, steroids)	Hypothyroidism: fatigue, mood disturbances, slowing of thought and speech, lethargy, bradycardia, cold sensitivity, weight gain, dry skin, hair loss, and menstrual irregularities Hyperthyroidism: fatigue, nervousness, anxiety, insomnia, tachycardia, tremor, heat intolerance, increased sweating, tachycardia, involuntary weight loss and (in case of Graves’ disease), eye symptoms (irritation, pain, discomfort in eye motion, diplopia)	Prominent fatigue with or without chronic widespread pain, mood and cognitive disturbances, insomnia, temperature sensitivity, tremor, weight change, and palpitation	↑ TSH (hypothyroidism) ↓ TSH (hyperthyroidism)
Obesity	Family inheritance, unhealthy diet, inactivity, drugs (antidepressants, antipsychotics, beta-blockers, steroids, insulin)	BMI ≥ 30 kg/m^2^, weakness, shortness of breath “adiposis dolorosa”	Chronic widespread pain, fatigue, exercise intolerance, sleep disturbances, “adiposis dolorosa” in patients with increased BMI	BMI calculation
**Infections**				
HIV	High-risk sexual behavior, intravenous drug use, needle-stick accidents, unsterile cutting or piercings, blood transfusions and organ transplants	Acquired immunodeficiency syndrome (AIDS)	Chronic widespread pain and/or fatigue in patients with high-risk sexual behavior, history of other sexually transmitted infections (e.g., syphilis, herpes, chlamydia, gonorrhea), recurrent infections, or lymphopenia	Positive HIV antibody test
HCV	High-risk sexual behavior, intravenous drug use, needle-stick accidents, unsterile cutting or piercings, blood transfusions and organ transplants	Acute or chronic hepatitis	Chronic widespread pain and/or fatigue in patients with high-risk sexual behavior, a history of other sexually transmitted infections (e.g., syphilis, herpes, chlamydia, gonorrhea)	Positive HCV antibody test
SARS-CoV-2	Close personal contact with infected individuals	COVID-19; long-COVID-19	Chronic widespread pain and/or fatigue with onset after SARS-CoV-2 infection	No specific tests available for post-COVID-19 conditions
Borrelia burgdorferi	Risk factors for tick bite exposure in an endemic area (e.g., gardening, hunting, or walking in high grasses)	Erythema migrans, neuroborreliosis, carditis, arthritis	Chronic widespread pain and/or fatigue in patients with a past history of tick bite or erythema migrans following potential exposure to tick bite	Positive Borrelia antibody test
**Others**				
Peripheral Neuropathy	Diabetes, alcohol abuse, malnutrition, monoclonal gammopathy, drugs (chemotherapy, amiodarone, colchicine, gold salts) heavy metals intoxication	Sensory (pins and needles, burning or sharp pain, numbness, decreased sensitivity to pain or temperature), motor (muscle weakness/paralysis; twitching or muscle cramps) or autonomic (sicca syndrome, accommodation problems, orthostatic hypotension, hypohidrosis or hyperhidrosis, hot flashes, intestinal problems, gastroparesis, palpitations) disturbances	Chronic widespread pain with prominent neuropathic features (burning, shooting, pricking, pins and needles, squeezing, or freezing pain) and/or accompanied by allodynia, hyperalgesia, paresthesia, weakness, imbalance, and/or autonomic dysfunction	Nerve conduction studies; skin biopsy (small fiber neuropathy)
Obstructive sleep apnea	Obesity, heart failure, acromegaly, hypothyroidism, smoking, craniofacial abnormalities	Daytime sleepiness, fatigue, non-refreshing sleep, snoring, paroxysmal nocturnal dyspnea, decreased concentration and memory, headache, nocturia	Chronic fatigue and/or widespread pain with snoring or prominent sleep disturbances (e.g., non-refreshing sleep) and daytime cognitive symptoms	Polysomnography
Celiac disease	Family history, type 1 diabetes, Hashimoto thyroiditis, microscopic colitis, Addison’s disease, Down syndrome, William syndrome, Turner syndrome	Abdominal pain, diarrhea or constipation, weight loss, bloating, nausea/vomiting	Chronic widespread pain and/or fatigue with prominent gastrointestinal symptoms, unexplained iron deficiency or low bone mass	Positive tTG-IgA
Cancer	Previous history of cancer, aging, smoking, obesity, alcohol abuse, infectious agents (e.g., HPV), radiation and/or chemical exposure, chronic inflammation, immunosuppression	Cancer-specific manifestations; bone metastasis; paraneoplastic syndromes (e.g., cancer-associated myositis)	Progressive, treatment-resistant fatigue/muscle weakness and/or multifocal bone pain in patients with known risk factors associated with organ-specific or constitutional symptoms or unexplained weight loss	No universal screening test is available; consider the possibility of further investigation based on demographic/epidemiological factors and specific symptoms
Drugs	Chronic treatment	-	Chronic widespread pain and/or fatigue in patients exposed to medications notoriously associated with musculoskeletal adverse events	Lipid-lowering drugs, bisphosphonates, aromatase inhibitors, isotretinoin, and others

Legend: BMI, body mass index; COVID-19, coronavirus disease 2019; FM, fibromyalgia; HCV, hepatitis C virus; HIV, human immunodeficiency virus; HPV, human papillomavirus; PPIs, proton pump inhibitors; TSAT, transferrin saturation; SARS-CoV-2, severe acute respiratory syndrome coronavirus 2; tTG-IgA, tissue transglutaminase IgA antibody; TSH, thyroid-stimulating hormone.

## References

[1] Macfarlane G.J., Kronisch C., Dean L.E., Atzeni F., Häuser W., Fluß E., Choy E., Kosek E., Amris K., Branco J. (2017). EULAR revised recommendations for the management of fibromyalgia. Ann. Rheum. Dis..

[2] Chinn S., Caldwell W., Gritsenko K. (2016). Fibromyalgia Pathogenesis and Treatment Options Update. Curr. Pain Headache Rep..

[3] Wolfe F., Clauw D.J., Fitzcharles M.-A., Goldenberg D.L., Häuser W., Katz R.L., Mease P.J., Russell A.S., Russell I.J., Walitt B. (2016). 2016 Revisions to the 2010/2011 fibromyalgia diagnostic criteria. Semin. Arthritis Rheum..

[4] Hunt A., Harrington D., Robinson S. (2014). Vitamin B12 deficiency. BMJ.

[5] Green R., Allen L.H., Bjørke-Monsen A.-L., Brito A., Guéant J.-L., Miller J.W., Molloy A.M., Nexo E., Stabler S., Toh B.-H. (2017). Vitamin B12 deficiency. Nat. Rev. Dis. Primer.

[6] Andrès E., Zulfiqar A.-A., Vogel T. (2020). State of the art review: Oral and nasal vitamin B12 therapy in the elderly. QJM Mon. J. Assoc. Phys..

[7] Pawlak R., Lester S.E., Babatunde T. (2014). The prevalence of cobalamin deficiency among vegetarians assessed by serum vitamin B12: A review of literature. Eur. J. Clin. Nutr..

[8] Salinas M., Flores E., López-Garrigós M., Leiva-Salinas C. (2018). Vitamin B12 deficiency and clinical laboratory: Lessons revisited and clarified in seven questions. Int. J. Lab. Hematol..

[9] Lindenbaum J., Healton E.B., Savage D.G., Brust J.C., Garrett T.J., Podell E.R., Marcell P.D., Stabler S.P., Allen R.H. (1988). Neuropsychiatric disorders caused by cobalamin deficiency in the absence of anemia or macrocytosis. N. Engl. J. Med..

[10] Kumar N. (2014). Neurologic aspects of cobalamin (B12) deficiency. Handb. Clin. Neurol..

[11] Devalia V., Hamilton M.S., Molloy A.M. (2014). British Committee for Standards in Haematology Guidelines for the diagnosis and treatment of cobalamin and folate disorders. Br. J. Haematol..

[12] Wolffenbuttel B.H.R., Wouters H.J.C.M., Heiner-Fokkema M.R., van der Klauw M.M. (2019). The Many Faces of Cobalamin (Vitamin B12) Deficiency. Mayo Clin. Proc. Innov. Qual. Outcomes.

[13] Gharibpoor F., Ghavidel-Parsa B., Sattari N., Bidari A., Nejatifar F., Montazeri A. (2022). Effect of vitamin B12 on the symptom severity and psychological profile of fibromyalgia patients; a prospective pre-post study. BMC Rheumatol..

[14] British Columbia Guidelines on Cobalamin (Vitamin B12) and Folate Deficiency. https://www2.gov.bc.ca/gov/content/health/practitioner-professional-resources/bc-guidelines/vitamin-b12.

[15] Holick M.F., Binkley N.C., Bischoff-Ferrari H.A., Gordon C.M., Hanley D.A., Heaney R.P., Murad M.H., Weaver C.M. (2011). Endocrine Society Evaluation, treatment, and prevention of vitamin D deficiency: An Endocrine Society clinical practice guideline. J. Clin. Endocrinol. Metab..

[16] Looker A.C., Johnson C.L., Lacher D.A., Pfeiffer C.M., Schleicher R.L., Sempos C.T. (2011). Vitamin D Status: United States, 2001–2006.

[17] Sprague S., Petrisor B., Scott T., Devji T., Phillips M., Spurr H., Bhandari M., Slobogean G.P. (2016). What Is the Role of Vitamin D Supplementation in Acute Fracture Patients? A Systematic Review and Meta-Analysis of the Prevalence of Hypovitaminosis D and Supplementation Efficacy. J. Orthop. Trauma.

[18] Książek A., Zagrodna A., Słowińska-Lisowska M. (2019). Vitamin D, Skeletal Muscle Function and Athletic Performance in Athletes-A Narrative Review. Nutrients.

[19] Wang T.-T., Tavera-Mendoza L.E., Laperriere D., Libby E., MacLeod N.B., Nagai Y., Bourdeau V., Konstorum A., Lallemant B., Zhang R. (2005). Large-scale in silico and microarray-based identification of direct 1,25-dihydroxyvitamin D3 target genes. Mol. Endocrinol..

[20] Neal S., Sykes J., Rigby M., Hess B. (2015). A review and clinical summary of vitamin D in regard to bone health and athletic performance. Phys. Sportsmed..

[21] Owens D.J., Allison R., Close G.L. (2018). Vitamin D and the Athlete: Current Perspectives and New Challenges. Sports Med. Auckl. NZ.

[22] Shipton E.E., Shipton E.A. (2015). Vitamin D Deficiency and Pain: Clinical Evidence of Low Levels of Vitamin D and Supplementation in Chronic Pain States. Pain Ther..

[23] Powell H.S., Greenberg D. (2006). Tackling vitamin D deficiency. Postgrad. Med..

[24] Ellis S.D., Kelly S.T., Shurlock J.H., Hepburn A.L.N. (2018). The role of vitamin D testing and replacement in fibromyalgia: A systematic literature review. BMC Rheumatol..

[25] Martins Y.A., Cardinali C.A.E.F., Ravanelli M.I., Brunaldi K. (2020). Is hypovitaminosis D associated with fibromyalgia? A systematic review. Nutr. Rev..

[26] Lopez A., Cacoub P., Macdougall I.C., Peyrin-Biroulet L. (2016). Iron deficiency anaemia. Lancet Lond. Engl..

[27] Camaschella C. (2015). Iron-deficiency anemia. N. Engl. J. Med..

[28] Stugiewicz M., Tkaczyszyn M., Kasztura M., Banasiak W., Ponikowski P., Jankowska E.A. (2016). The influence of iron deficiency on the functioning of skeletal muscles: Experimental evidence and clinical implications. Eur. J. Heart Fail..

[29] Pamuk G.E., Pamuk O.N., Set T., Harmandar O., Yeşil N. (2008). An increased prevalence of fibromyalgia in iron deficiency anemia and thalassemia minor and associated factors. Clin. Rheumatol..

[30] Hamarat H., Gürcü S., Kıvanç B.K., Aydemir A.E. (2023). Ferric carboxymaltose therapy reduces pain and improves the quality of life in female patients with fibromyalgia. Eur. Rev. Med. Pharmacol. Sci..

[31] Yao W.-C., Chen H.-J., Leong K.-H., Chang K.-L., Wang Y.-T.T., Wu L.-C., Tung P.-Y., Kuo C.-F., Lin C.-C., Tsai S.-Y. (2021). The risk of fibromyalgia in patients with iron deficiency anemia: A nationwide population-based cohort study. Sci. Rep..

[32] Beard J.L., Connor J.R., Jones B.C. (2009). Iron in the Brain. Nutr. Rev..

[33] Snook J., Bhala N., Beales I.L.P., Cannings D., Kightley C., Logan R.P., Pritchard D.M., Sidhu R., Surgenor S., Thomas W. (2021). British Society of Gastroenterology guidelines for the management of iron deficiency anaemia in adults. Gut.

[34] Pavord S., Daru J., Prasannan N., Robinson S., Stanworth S., Girling J. (2020). BSH Committee UK guidelines on the management of iron deficiency in pregnancy. Br. J. Haematol..

[35] Wolf F.I., Cittadini A. (2003). Chemistry and biochemistry of magnesium. Mol. Aspects Med..

[36] Pasternak K., Kocot J., Horecka A. (2012). Biochemistry of magnesium. J. Elem..

[37] Castiglioni S., Maier J.A.M. (2011). Magnesium and cancer: A dangerous liason. Magnes. Res..

[38] Larsson S.C., Bergkvist L., Wolk A. (2005). Magnesium intake in relation to risk of colorectal cancer in women. JAMA.

[39] Chakraborti S., Chakraborti T., Mandal M., Mandal A., Das S., Ghosh S. (2002). Protective role of magnesium in cardiovascular diseases: A review. Mol. Cell. Biochem..

[40] Shin H.-J., Na H.-S., Do S.-H. (2020). Magnesium and Pain. Nutrients.

[41] Gröber U., Schmidt J., Kisters K. (2015). Magnesium in Prevention and Therapy. Nutrients.

[42] Saris N.E., Mervaala E., Karppanen H., Khawaja J.A., Lewenstam A. (2000). Magnesium. An update on physiological, clinical and analytical aspects. Clin. Chim. Acta Int. J. Clin. Chem..

[43] Grzebisz W. (2011). Magnesium—Food and human health. J. Elemntology.

[44] Boulis M., Boulis M., Clauw D. (2021). Magnesium and Fibromyalgia: A Literature Review. J. Prim. Care Community Health.

[45] Blaszczyk U., Duda-Chodak A. (2013). Magnesium: Its role in nutrition and carcinogenesis. Rocz. Panstw. Zakl. Hig..

[46] Andretta A., Dias Batista E., Madalozzo Schieferdecker M.E., Rasmussen Petterle R., Boguszewski C.L., Dos Santos Paiva E. (2019). Relation between magnesium and calcium and parameters of pain, quality of life and depression in women with fibromyalgia. Adv. Rheumatol. Lond. Engl..

[47] Macian N., Dualé C., Voute M., Leray V., Courrent M., Bodé P., Giron F., Sonneville S., Bernard L., Joanny F. (2022). Short-Term Magnesium Therapy Alleviates Moderate Stress in Patients with Fibromyalgia: A Randomized Double-Blind Clinical Trial. Nutrients.

[48] Kisters K. (2013). What is the correct magnesium supplement?. Magnes. Res..

[49] Léger D. (2008). Scurvy: Reemergence of nutritional deficiencies. Can. Fam. Phys. Med. Fam. Can..

[50] Weinstein M., Babyn P., Zlotkin S. (2001). An orange a day keeps the doctor away: Scurvy in the year 2000. Pediatrics.

[51] Carr A.C., McCall C. (2017). The role of vitamin C in the treatment of pain: New insights. J. Transl. Med..

[52] Fain O. (2005). Musculoskeletal manifestations of scurvy. Jt. Bone Spine.

[53] Aïm F., Klouche S., Frison A., Bauer T., Hardy P. (2017). Efficacy of vitamin C in preventing complex regional pain syndrome after wrist fracture: A systematic review and meta-analysis. Orthop. Traumatol. Surg. Res..

[54] Chaker L., Bianco A.C., Jonklaas J., Peeters R.P. (2017). Hypothyroidism. Lancet Lond. Engl..

[55] Taylor P.N., Albrecht D., Scholz A., Gutierrez-Buey G., Lazarus J.H., Dayan C.M., Okosieme O.E. (2018). Global epidemiology of hyperthyroidism and hypothyroidism. Nat. Rev. Endocrinol..

[56] Persani L., Brabant G., Dattani M., Bonomi M., Feldt-Rasmussen U., Fliers E., Gruters A., Maiter D., Schoenmakers N., van Trotsenburg A.S.P. (2018). 2018 European Thyroid Association (ETA) Guidelines on the Diagnosis and Management of Central Hypothyroidism. Eur. Thyroid J..

[57] Garber J.R., Cobin R.H., Gharib H., Hennessey J.V., Klein I., Mechanick J.I., Pessah-Pollack R., Singer P.A., Woeber K.A. (2012). American Association Of Clinical Endocrinologists and American Thyroid Association Taskforce on Hypothyroidism in Adults Clinical practice guidelines for hypothyroidism in adults: Cosponsored by the American Association of Clinical Endocrinologists and the American Thyroid Association. Thyroid Off. J. Am. Thyroid Assoc..

[58] Ross D.S., Burch H.B., Cooper D.S., Greenlee M.C., Laurberg P., Maia A.L., Rivkees S.A., Samuels M., Sosa J.A., Stan M.N. (2016). 2016 American Thyroid Association Guidelines for Diagnosis and Management of Hyperthyroidism and Other Causes of Thyrotoxicosis. Thyroid Off. J. Am. Thyroid Assoc..

[59] Franklyn J.A., Boelaert K. (2012). Thyrotoxicosis. Lancet Lond. Engl..

[60] Smith T.J., Hegedüs L. (2016). Graves’ Disease. N. Engl. J. Med..

[61] Bahn R.S. (2010). Graves’ ophthalmopathy. N. Engl. J. Med..

[62] Smith T.J., Janssen J.A.M.J.L. (2019). Insulin-like Growth Factor-I Receptor and Thyroid-Associated Ophthalmopathy. Endocr. Rev..

[63] De Leo S., Lee S.Y., Braverman L.E. (2016). Hyperthyroidism. Lancet Lond. Engl..

[64] Park S., Kwon J.-S., Park Y.-B., Park J.W. (2022). Is thyroid autoimmunity a predisposing factor for fibromyalgia? A systematic review and meta-analysis. Clin. Exp. Rheumatol..

[65] Haliloglu S., Ekinci B., Uzkeser H., Sevimli H., Carlioglu A., Macit P.M. (2017). Fibromyalgia in patients with thyroid autoimmunity: Prevalence and relationship with disease activity. Clin. Rheumatol..

[66] Garvey W.T., Mechanick J.I., Brett E.M., Garber A.J., Hurley D.L., Jastreboff A.M., Nadolsky K., Pessah-Pollack R., Plodkowski R. (2016). Reviewers of the AACE/ACE Obesity Clinical Practice Guidelines American Association of Clinical Endocrinologists and American College of Endocrinology Comprehensive Clinical Practice Guidelines for Medical Care of Patients with Obesity. Endocr. Pract. Off. J. Am. Coll. Endocrinol. Am. Assoc. Clin. Endocrinol..

[67] Bray G.A., Frühbeck G., Ryan D.H., Wilding J.P.H. (2016). Management of obesity. Lancet.

[68] (2016). NCD Risk Factor Collaboration (NCD-RisC) Trends in adult body-mass index in 200 countries from 1975 to 2014: A pooled analysis of 1698 population-based measurement studies with 19·2 million participants. Lancet Lond. Engl..

[69] Hales C.M., Fryar C.D., Carroll M.D., Freedman D.S., Aoki Y., Ogden C.L. (2018). Differences in Obesity Prevalence by Demographic Characteristics and Urbanization Level Among Adults in the United States, 2013-2016. JAMA.

[70] Rubino F., Puhl R.M., Cummings D.E., Eckel R.H., Ryan D.H., Mechanick J.I., Nadglowski J., Ramos Salas X., Schauer P.R., Twenefour D. (2020). Joint international consensus statement for ending stigma of obesity. Nat. Med..

[71] Yu E., Ley S.H., Manson J.E., Willett W., Satija A., Hu F.B., Stokes A. (2017). Weight History and All-Cause and Cause-Specific Mortality in Three Prospective Cohort Studies. Ann. Intern. Med..

[72] Yancy W.S., Olsen M.K., Westman E.C., Bosworth H.B., Edelman D. (2002). Relationship between obesity and health-related quality of life in men. Obes. Res..

[73] Kucharz E.J., Kopeć-Mędrek M., Kramza J., Chrzanowska M., Kotyla P. (2019). Dercum’s disease (adiposis dolorosa): A review of clinical presentation and management. Reumatologia.

[74] Ursini F., Naty S., Grembiale R.D. (2011). Fibromyalgia and obesity: The hidden link. Rheumatol. Int..

[75] D’Onghia M., Ciaffi J., Lisi L., Mancarella L., Ricci S., Stefanelli N., Meliconi R., Ursini F. (2021). Fibromyalgia and obesity: A comprehensive systematic review and meta-analysis. Semin. Arthritis Rheum..

[76] Ciaffi J., Lisi L., Mari A., Mancarella L., Brusi V., Pignatti F., Ricci S., Vitali G., Stefanelli N., Assirelli E. (2023). Efficacy, safety and tolerability of very low-calorie ketogenic diet in obese women with fibromyalgia: A pilot interventional study. Front. Nutr..

[77] Epstein L.J., Kristo D., Strollo P.J., Friedman N., Malhotra A., Patil S.P., Ramar K., Rogers R., Schwab R.J., Weaver E.M. (2009). Clinical guideline for the evaluation, management and long-term care of obstructive sleep apnea in adults. J. Clin. Sleep Med. JCSM Off. Publ. Am. Acad. Sleep Med..

[78] Jordan A.S., McSharry D.G., Malhotra A. (2014). Adult obstructive sleep apnoea. Lancet Lond. Engl..

[79] Lévy P., Kohler M., McNicholas W.T., Barbé F., McEvoy R.D., Somers V.K., Lavie L., Pépin J.-L. (2015). Obstructive sleep apnoea syndrome. Nat. Rev. Dis. Primer.

[80] Younes M. (2019). Pathogenesis of Obstructive Sleep Apnea. Clin. Chest Med..

[81] Greenstone M., Hack M. (2014). Obstructive sleep apnoea. BMJ.

[82] Rosenfeld V.W., Rutledge D.N., Stern J.M. (2015). Polysomnography with quantitative EEG in patients with and without fibromyalgia. J. Clin. Neurophysiol. Off. Publ. Am. Electroencephalogr. Soc..

[83] Meresh E.S., Artin H., Joyce C., Birch S., Daniels D., Owens J.H., La Rosa A.J., Rao M.S., Halaris A. (2019). Obstructive sleep apnea co-morbidity in patients with fibromyalgia: A single-center retrospective analysis and literature review. Open Access Rheumatol. Res. Rev..

[84] Sateia M.J. (2014). International classification of sleep disorders-third edition: Highlights and modifications. Chest.

[85] Tanna N., Smith B.D., Zapanta P.E., Karanetz I., Andrews B.T., Urata M.M., Bradley J.P. (2016). Surgical Management of Obstructive Sleep Apnea. Plast. Reconstr. Surg..

[86] Feltner C., Wallace I.F., Aymes S., Cook Middleton J., Hicks K.L., Schwimmer M., Baker C., Balio C.P., Moore D., Voisin C.E. (2022). Screening for Obstructive Sleep Apnea in Adults: Updated Evidence Report and Systematic Review for the US Preventive Services Task Force. JAMA.

[87] Tomfohr L.M., Ancoli-Israel S., Loredo J.S., Dimsdale J.E. (2011). Effects of continuous positive airway pressure on fatigue and sleepiness in patients with obstructive sleep apnea: Data from a randomized controlled trial. Sleep.

[88] Borsook D. (2012). Neurological diseases and pain. Brain J. Neurol..

[89] Azhary H., Farooq M.U., Bhanushali M., Majid A., Kassab M.Y. (2010). Peripheral neuropathy: Differential diagnosis and management. Am. Fam. Phys..

[90] Hanewinckel R., Drenthen J., van Oijen M., Hofman A., van Doorn P.A., Ikram M.A. (2016). Prevalence of polyneuropathy in the general middle-aged and elderly population. Neurology.

[91] Barrell K., Smith A.G. (2019). Peripheral Neuropathy. Med. Clin. N. Am..

[92] Callaghan B.C., Price R.S., Feldman E.L. (2015). Distal Symmetric Polyneuropathy: A Review. JAMA.

[93] Alport A.R., Sander H.W. (2012). Clinical approach to peripheral neuropathy: Anatomic localization and diagnostic testing. Contin. Minneap. Minn.

[94] Raasing L.R.M., Vogels O.J.M., Veltkamp M., van Swol C.F.P., Grutters J.C. (2021). Current View of Diagnosing Small Fiber Neuropathy. J. Neuromuscul. Dis..

[95] Grayston R., Czanner G., Elhadd K., Goebel A., Frank B., Üçeyler N., Malik R.A., Alam U. (2019). A systematic review and meta-analysis of the prevalence of small fiber pathology in fibromyalgia: Implications for a new paradigm in fibromyalgia etiopathogenesis. Semin. Arthritis Rheum..

[96] Bailly F. (2021). The challenge of differentiating fibromyalgia from small-fiber neuropathy in clinical practice. Jt. Bone Spine.

[97] Beynon A.M., Hebert J.J., Hodgetts C.J., Boulos L.M., Walker B.F. (2020). Chronic physical illnesses, mental health disorders, and psychological features as potential risk factors for back pain from childhood to young adulthood: A systematic review with meta-analysis. Eur. Spine J..

[98] Warren J.W., Brown V., Jacobs S., Horne L., Langenberg P., Greenberg P. (2008). Urinary Tract Infection and Inflammation at Onset of Interstitial Cystitis/Painful Bladder Syndrome. Urology.

[99] Hickie I., Davenport T., Wakefield D., Vollmer-Conna U., Cameron B., Vernon S.D., Reeves W.C., Lloyd A. (2006). Post-infective and chronic fatigue syndromes precipitated by viral and non-viral pathogens: Prospective cohort study. BMJ.

[100] Kallio-Laine K., Seppänen M., Lokki M.-L., Lappalainen M., Notkola I.-L., Seppälä I., Koskinen M., Valtonen V., Kalso E. (2008). Widespread unilateral pain associated with herpes simplex virus infections. J. Pain.

[101] Navis A., Jiao J., George M.C., Simpson D., Robinson-Papp J. (2018). Comorbid Pain Syndromes in HIV-Associated Peripheral Neuropathy. Pain Med..

[102] Madden V.J., Parker R., Goodin B.R. (2020). Chronic pain in people with HIV: A common comorbidity and threat to quality of life. Pain Manag..

[103] European Association for the Study of the Liver (2018). Electronic address: Easloffice@easloffice.eu; European Association for the Study of the Liver EASL Recommendations on Treatment of Hepatitis C 2018. J. Hepatol..

[104] Moretti R., Caruso P., Dal Ben M., Gazzin S., Tiribelli C. (2018). Hepatitis C-related cryoglobulinemic neuropathy: Potential role of oxcarbazepine for pain control. BMC Gastroenterol..

[105] Cacoub P., Saadoun D. (2021). Extrahepatic Manifestations of Chronic HCV Infection. N. Engl. J. Med..

[106] Fernandez-de-Las-Peñas C., Notarte K.I., Macasaet R., Velasco J.V., Catahay J.A., Ver A.T., Chung W., Valera-Calero J.A., Navarro-Santana M. (2024). Persistence of post-COVID symptoms in the general population two years after SARS-CoV-2 infection: A systematic review and meta-analysis. J. Infect..

[107] Ursini F., Ciaffi J., Mancarella L., Lisi L., Brusi V., Cavallari C., D’Onghia M., Mari A., Borlandelli E., Faranda Cordella J. (2021). Fibromyalgia: A new facet of the post-COVID-19 syndrome spectrum? Results from a web-based survey. RMD Open.

[108] Ursini F., Ruscitti P., D’Angelo S., Cacciapaglia F., De Angelis R., Campochiaro C., Caso F., De Santis M., Di Cola I., Parisi S. (2021). Broad clinical spectrum of SARS-CoV-2-associated inflammatory joint disease in adults: A report of 35 cases from the COVID-19 & Autoimmune Systemic Disease Italian study group. Ann. Rheum. Dis..

[109] Ursini F., Ruscitti P., Addimanda O., Foti R., Raimondo V., Murdaca G., Caira V., Pigatto E., Cuomo G., Lo Gullo A. (2023). Inflammatory rheumatic diseases with onset after SARS-CoV-2 infection or COVID-19 vaccination: A report of 267 cases from the COVID-19 and ASD group. RMD Open.

[110] Ciaffi J., Vanni E., Mancarella L., Brusi V., Lisi L., Pignatti F., Naldi S., Assirelli E., Neri S., Reta M. (2023). Post-Acute COVID-19 Joint Pain and New Onset of Rheumatic Musculoskeletal Diseases: A Systematic Review. Diagnostics.

[111] Ursini F., Ruscitti P., Raimondo V., De Angelis R., Cacciapaglia F., Pigatto E., Olivo D., Di Cola I., Galluccio F., Francioso F. (2022). Systemic syndromes of rheumatological interest with onset after COVID-19 vaccine administration: A report of 30 cases. Clin. Rheumatol..

[112] Lantos P.M., Rumbaugh J., Bockenstedt L.K., Falck-Ytter Y.T., Aguero-Rosenfeld M.E., Auwaerter P.G., Baldwin K., Bannuru R.R., Belani K.K., Bowie W.R. (2021). Clinical Practice Guidelines by the Infectious Diseases Society of America (IDSA), American Academy of Neurology (AAN), and American College of Rheumatology (ACR): 2020 Guidelines for the Prevention, Diagnosis and Treatment of Lyme Disease. Clin. Infect. Dis. Off. Publ. Infect. Dis. Soc. Am..

[113] Stanek G., Wormser G.P., Gray J., Strle F. (2012). Lyme borreliosis. Lancet Lond. Engl..

[114] Dinerman H., Steere A.C. (1992). Lyme disease associated with fibromyalgia. Ann. Intern. Med..

[115] WHO Fact SHeets. https://www.who.int/health-topics/cancer#tab=tab_1.

[116] Mercadante S. (2022). Cancer Pain Treatment Strategies in Patients with Cancer. Drugs.

[117] Lawrence D.P., Kupelnick B., Miller K., Devine D., Lau J. (2004). Evidence report on the occurrence, assessment, and treatment of fatigue in cancer patients. J. Natl. Cancer Inst. Monogr..

[118] McBeth J., Symmons D.P., Silman A.J., Allison T., Webb R., Brammah T., Macfarlane G.J. (2008). Musculoskeletal pain is associated with a long-term increased risk of cancer and cardiovascular-related mortality. Rheumatology.

[119] Koo M.M., Swann R., McPhail S., Abel G.A., Elliss-Brookes L., Rubin G.P., Lyratzopoulos G. (2020). Presenting symptoms of cancer and stage at diagnosis: Evidence from a cross-sectional, population-based study. Lancet Oncol..

[120] Prabhakar A., Smith T.J. (2021). Total Pain #417. J. Palliat. Med..

[121] Silvestris F., Lombardi L., De Matteo M., Bruno A., Dammacco F. (2007). Myeloma bone disease: Pathogenetic mechanisms and clinical assessment. Leuk. Res..

[122] Sousa S., Clézardin P. (2018). Bone-Targeted Therapies in Cancer-Induced Bone Disease. Calcif. Tissue Int..

[123] Kim W., Han I., Kang S., Lee S.A., Kim H.-S. (2014). Non-spine bone metastasis as an initial manifestation of cancer in Korea. J. Korean Med. Sci..

[124] Falk S., Bannister K., Dickenson A.H. (2014). Cancer pain physiology. Br. J. Pain.

[125] Kan C., Vargas G., Pape F.L., Clézardin P. (2016). Cancer Cell Colonisation in the Bone Microenvironment. Int. J. Mol. Sci..

[126] Panaroni C., Yee A.J., Raje N.S. (2017). Myeloma and Bone Disease. Curr. Osteoporos. Rep..

[127] Minisola S., Pepe J., Piemonte S., Cipriani C. (2015). The diagnosis and management of hypercalcaemia. BMJ.

[128] Zhan Y., Zhang G., Li M., Zhou X. (2021). Whole-Body MRI vs. PET/CT for the Detection of Bone Metastases in Patients With Prostate Cancer: A Systematic Review and Meta-Analysis. Front. Oncol..

[129] Christensen J.F., Jones L.W., Andersen J.L., Daugaard G., Rorth M., Hojman P. (2014). Muscle dysfunction in cancer patients. Ann. Oncol. Off. J. Eur. Soc. Med. Oncol..

[130] Oldroyd A.G.S., Allard A.B., Callen J.P., Chinoy H., Chung L., Fiorentino D., George M.D., Gordon P., Kolstad K., Kurtzman D.J.B. (2021). A systematic review and meta-analysis to inform cancer screening guidelines in idiopathic inflammatory myopathies. Rheumatol. Oxf. Engl..

[131] Akkaya N., Atalay N.S., Selcuk S.T., Alkan H., Catalbas N., Sahin F. (2013). Frequency of fibromyalgia syndrome in breast cancer patients. Int. J. Clin. Oncol..

[132] Caio G., Volta U., Sapone A., Leffler D.A., De Giorgio R., Catassi C., Fasano A. (2019). Celiac disease: A comprehensive current review. BMC Med..

[133] Therrien A., Kelly C.P., Silvester J.A. (2020). Celiac Disease: Extraintestinal Manifestations and Associated Conditions. J. Clin. Gastroenterol..

[134] Rodrigo L., Blanco I., Bobes J., de Serres F.J. (2013). Remarkable prevalence of coeliac disease in patients with irritable bowel syndrome plus fibromyalgia in comparison with those with isolated irritable bowel syndrome: A case-finding study. Arthritis Res. Ther..

[135] Lebwohl B., Sanders D.S., Green P.H.R. (2018). Coeliac disease. Lancet Lond. Engl..

[136] Laurikka P., Salmi T., Collin P., Huhtala H., Mäki M., Kaukinen K., Kurppa K. (2016). Gastrointestinal Symptoms in Celiac Disease Patients on a Long-Term Gluten-Free Diet. Nutrients.

[137] Sansotta N., Amirikian K., Guandalini S., Jericho H. (2018). Celiac Disease Symptom Resolution: Effectiveness of the Gluten-free Diet. J. Pediatr. Gastroenterol. Nutr..

[138] Insani W.N., Whittlesea C., Alwafi H., Man K.K.C., Chapman S., Wei L. (2021). Prevalence of adverse drug reactions in the primary care setting: A systematic review and meta-analysis. PLoS ONE.

[139] Conforti A., Chiamulera C., Moretti U., Colcera S., Fumagalli G., Leone R. (2007). Musculoskeletal adverse drug reactions: A review of literature and data from ADR spontaneous reporting databases. Curr. Drug Saf..

[140] Mammen A.L. (2022). Statin-Associated Myalgias and Muscle Injury-Recognizing and Managing Both While Still Lowering the Low-Density Lipoprotein. Rheum. Dis. Clin. N. Am..

[141] (2022). Cholesterol Treatment Trialists’ Collaboration Effect of statin therapy on muscle symptoms: An individual participant data meta-analysis of large-scale, randomised, double-blind trials. Lancet Lond. Engl..

[142] Akimoto H., Negishi A., Oshima S., Okita M., Numajiri S., Inoue N., Ohshima S., Kobayashi D. (2018). Onset timing of statin-induced musculoskeletal adverse events and concomitant drug-associated shift in onset timing of MAEs. Pharmacol. Res. Perspect..

[143] D’Souza R.S., Whipple M.O., Vincent A. (2021). Statin Therapy and Symptom Burden in Patients With Fibromyalgia: A Prospective Questionnaire Study. Mayo Clin. Proc. Innov. Qual. Outcomes.

[144] Tomaszewski M., Stępień K.M., Tomaszewska J., Czuczwar S.J. (2011). Statin-induced myopathies. Pharmacol. Rep. PR.

[145] Simard C., Poirier P. (2006). Ezetimibe-associated myopathy in monotherapy and in combination with a 3-hydroxy-3-methylglutaryl coenzyme A reductase inhibitor. Can. J. Cardiol..

[146] Molokhia M., McKeigue P., Curcin V., Majeed A. (2008). Statin induced myopathy and myalgia: Time trend analysis and comparison of risk associated with statin class from 1991-2006. PLoS ONE.

[147] Ding L., Chen C., Yang Y., Fang J., Cao L., Liu Y. (2022). Musculoskeletal Adverse Events Associated with PCSK9 Inhibitors: Disproportionality Analysis of the FDA Adverse Event Reporting System. Cardiovasc. Ther..

[148] Jacobson T.A., Zimmerman F.H. (2006). Fibrates in combination with statins in the management of dyslipidemia. J. Clin. Hypertens. Greenwich Conn..

[149] Sieber P., Lardelli P., Kraenzlin C.A., Kraenzlin M.E., Meier C. (2013). Intravenous bisphosphonates for postmenopausal osteoporosis: Safety profiles of zoledronic acid and ibandronate in clinical practice. Clin. Drug Investig..

[150] Bock O., Boerst H., Thomasius F.E., Degner C., Stephan-Oelkers M., Valentine S.M., Felsenberg D. (2007). Common musculoskeletal adverse effects of oral treatment with once weekly alendronate and risedronate in patients with osteoporosis and ways for their prevention. J. Musculoskelet. Neuronal Interact..

[151] Wysowski D.K., Chang J.T. (2005). Alendronate and risedronate: Reports of severe bone, joint, and muscle pain. Arch. Intern. Med..

[152] Johnston S.R.D., Dowsett M. (2003). Aromatase inhibitors for breast cancer: Lessons from the laboratory. Nat. Rev. Cancer.

[153] Henry N.L., Giles J.T., Ang D., Mohan M., Dadabhoy D., Robarge J., Hayden J., Lemler S., Shahverdi K., Powers P. (2008). Prospective characterization of musculoskeletal symptoms in early stage breast cancer patients treated with aromatase inhibitors. Breast Cancer Res. Treat..

[154] Beckwée D., Leysen L., Meuwis K., Adriaenssens N. (2017). Prevalence of aromatase inhibitor-induced arthralgia in breast cancer: A systematic review and meta-analysis. Support. Care Cancer Off. J. Multinatl. Assoc. Support. Care Cancer.

[155] Vallerand I.A., Lewinson R.T., Farris M.S., Sibley C.D., Ramien M.L., Bulloch A.G.M., Patten S.B. (2018). Efficacy and adverse events of oral isotretinoin for acne: A systematic review. Br. J. Dermatol..

[156] Kaplan G., Haettich B. (1991). Rheumatological symptoms due to retinoids. Baillieres Clin. Rheumatol..

[157] Kapała J., Lewandowska J., Placek W., Owczarczyk-Saczonek A. (2022). Adverse Events in Isotretinoin Therapy: A Single-Arm Meta-Analysis. Int. J. Environ. Res. Public. Health.

[158] Bannwarth B. (2007). Drug-induced musculoskeletal disorders. Drug Saf..

